# The Characteristics and Outcomes of Patients Transported by Ambulance Due to Ambulatory Care Sensitive Condition: A Population-Based Descriptive Study in Osaka, Japan

**DOI:** 10.3389/fpubh.2022.911675

**Published:** 2022-06-30

**Authors:** Yusuke Katayama, Atsushi Kanehara, Yuya Yamashita, Tetsuhisa Kitamura, Jun Oda

**Affiliations:** ^1^Department of Traumatology and Acute Critical Medicine, Osaka University Graduate School of Medicine, Suita, Japan; ^2^Division of Environmental Medicine and Population Sciences, Department of Social and Environmental Medicine, Osaka University Graduate School of Medicine, Suita, Japan

**Keywords:** ambulatory care sensitive condition, emergency medicine, public health, epidemiology, primary care

## Abstract

**Background:**

Ambulatory care sensitive conditions (ACSCs) are defined as clinical conditions for which the risk of emergency hospital admission can be reduced by timely and effective ambulatory care. However, the actual status of patients with ACSCs who are transported by ambulance and their outcomes have not been fully elucidated. The purpose of this study was to reveal characteristics and outcomes of patients with ACSCs who were transported by ambulance using population-based registry data in Osaka, Japan.

**Methods:**

This descriptive epidemiological study was conducted in the 5-year period from January 2016 to December 2020, and included patients who were transported by ambulance due to sudden illness. In this study, ACSC was further classified into acute ACSCs, chronic ACSCs, and preventable ACSCs based on the ICD-10. The number of patients transported by ambulance for ACSCs per 100,000 population in each age group was calculated for each year. In addition, Poisson regression models were used to assess the trend in the number of ACSCs patients transported by ambulance.

**Results:**

A total of 1,572,152 patients were included in this study (acute ACSCs, *n* = 69,621; chronic ACSCs, *n* = 12,250; preventable ACSCs, *n* = 96,036; and non-ACSCs, *n* = 1,394,245). The overall median age was 71 years (95% confidence interval [CI], 46-92). Patients with acute ACSCs (median age: 76 years [IQR: 60-84]) and chronic ACSCs (median age: 80 years [IQR: 72-87]) were older, while patients with preventable ACSCs were younger (median age: 66 years [95%CI: 3-81]) and included a high proportion of children (33.3%, 32,002/96,036). Regarding the outcome after treatment at the emergency department, 42.6% (670,392/1,572.152) of patients were hospitalized, while 82.3% (10,079/12,250) of patients with chronic ACSCs were hospitalized. No change was observed in adults (*P* = 0.001) or elderly (age ≥65 years) individuals (*P* = 0.376) with preventable ACSCs, however, among children, the number increased until 2019 (732.5) and then decreased in 2020 (371.8) (*P* = 0.392).

**Conclusion:**

In this study, patients with chronic ACSCs were predominantly elderly, while patients with preventable ACSCs were polarized between children and the elderly. Among patients with preventable ACSCs, there was no change over time in adults and children, but there was a marked decrease among the elderly after 2020.

## Introduction

Internationally, national health systems are facing the problem of an increasing number of elderly individuals who require nursing care (1). On the other hand, emergency medical systems are public services that are used all over the world. As the number of elderly individuals who require nursing care and medical treatment increases, so does the demand for emergency medical services (EMS) when the condition of elderly individuals suddenly deteriorates. In Japan, the elderly population is continuing to increase, and the number of ambulance dispatches and number of patients transported by ambulance is continuing to increase; at present, the number of patients transported by ambulance amounts to 5.3 million per year (2). However, the number of available hospital beds has not increased in order control the ever-increasing medical costs (3). Consequently, the number of hospital beds in emergency medical institutions is always limited, and there is a social problem in relation to difficulty in hospital acceptance of these emergency patients (4).

In the 1990s, ambulatory care sensitive conditions (ACSCs) were defined as clinical conditions for which the risk of emergency hospital admission can be reduced by timely and effective ambulatory care (5). Ambulatory care mainly means primary care community services, and outpatient care. These conditions are used as indicators for local healthcare systems. For example, if patients with chronic diseases and the elderly receive appropriate treatment in a timely manner in outpatient clinics and hospitals, there may be fewer ambulance transportations and hospitalizations for these patients. However, the actual status of patients with ACSCs who are transported by ambulance and their outcomes have not been revealed in Japan.

In Osaka prefecture, a population-based patient registry (ORION) for all patients transported by ambulance is in operation, and data on all patients transported by ambulance in Osaka Prefecture from ambulance dispatch to hospital discharge have been continuously collected since 2015 (6, 7). The purpose of this study was to reveal the characteristics and outcomes of patients with ACSCs who are transported by ambulance using the ORION registry data.

## Materials and Methods

### Study Design, Settings

This descriptive epidemiological study was conducted in Osaka Prefecture in the 5-year period from January 2016 to December 2020. Osaka Prefecture is the largest metropolitan area in western Japan, with 8.8 million people living in an area of 1,905 km^2^ (8). This study included patients who were transported by ambulance due to sudden illness. Patients who were transported by ambulance due to non-sudden illness (e.g., traffic accidents or industrial accidents), patients who were not transported to clinics and hospitals, and patients who were transported to hospital and clinics outside Osaka Prefecture were excluded from the analysis. This study was approved by the ethics committees of Osaka University Graduate School of Medicine (Approval No. 15003; Suita, Japan). Because the ORION data are anonymized without specific personal data (e.g., patient name and address), the requirement of informed consent was waived. This report was written in accordance with the STROBE statement on cohort and cross-sectional studies (9).

### The ORION System

We previously described the ORION system in detail (6, 7). EMS personnel at the scene operate the ORION smartphone app in order to search for an appropriate hospital to admit for emergency patients. All of the data input into the smartphone app, such as systolic blood pressure, heart rate, and time of the call to the hospital for acceptance, are recorded. The smartphone app data are accumulated in the ORION cloud server, and in cooperation with the dispatched EMS personnel, data managers at each fire department directly input or upload the ambulance record of each emergency patient so that it can be connected with the smartphone app data. Then, the staff of each emergency hospital also directly input or upload the patient's data (e.g., the diagnosis and prognosis). The results of the data aggregated in the ORION system are fed back to each fire department and emergency hospital. The Department of Public Health of Osaka Prefecture can also research the situation of the emergency medical system and analyze the effects of health policy on the emergency medical system using these data. The ORION system has been in place in all fire departments and emergency hospitals in Osaka Prefecture since January 2016.

### ACSCs in This Study

ACSCs have already been defined in a previous study (5). In the present study, ACSC was further classified into acute ACSCs, chronic ACSCs, and preventable ACSCs based on the ICD-10. Acute ACSCs were defined as acute diseases (e.g., volume depletion and gastric ulcer) with the following ICD-10 codes: A69, E40, E41, E42, E43, E55, E86, H66, H67, J02, J03, J06, J31.2, K02, K03, K04, K05, K06, K08, K09.9, K12, K13, K25, K26, K27, K28, K52.2-9, L03, L04, L08, L88, L98, N10, N11, N12, N13.6, N39, N70, N73, and R02. Chronic ACSCs were defined as chronically managed diseases (e.g., convulsions and heart failure), with the following ICD-10 codes: D50, E10, E11, E12, E13, E14, G40, G41, I10, I11, I20, I24, I50, J20, J41, J42, J43, J44, J45, J46, J47, J81, and R56. Preventable ACSCs were defined as vaccine-preventable infectious diseases (e.g., influenza and mumps) with the following ICD-10 codes: A15, A16, A19, A35, A36, A37, A80, B05, B06, B169, B18, B26, G20, J10, J11, J13, J14, J15.3, J15.4, J15.7, J15.9, J16.8, J18.1, and J18.8.

### Statistical Analysis

Data were presented as the median and interquartile range (IQR) for continuous variables, and the actual number and percentage for categorical variables. Age groups were classified as follows: child (0–19 years), adult (20–64 years), and elderly (≥65 years). The number of patients transported by ambulance for ACSCs per 100,000 population in each age group was calculated for each year. Because we assumed that ACSC patients transported by ambulance were rare, Poisson regression models were applied to assess the trend in the number of ACSCs patients transported by ambulance. The time of day when the ambulance was requested was classified as daytime (9:00–17:59) or nighttime (0:00–8:59, 18:00–23:59). All statistical analyses were performed using the SPSS software program (ver.27.0 J, IBM Corp, Armonk, New York).

## Results

A total of 1,572,152 patients were included in this study (acute ACSCs, *n* = 69,621; chronic ACSCs, *n* = 12,250; preventable ACSCs, *n* = 96,036; and non-ACSCs, *n* = 1,394,245). Table 1 shows the characteristics and outcomes of patients who were transported by ambulance in this study. The overall median age was 71 years [95% confidence interval (CI), 46–92]. Patients with acute ACSCs [median age: 76 years (IQR: 60–84)] and chronic ACSCs [median age: 80 years (IQR: 72–87)] were older, while patients with preventable ACSCs were younger [median age: 66 years (95%CI: 3–81)] and included a high proportion of children (33.3%, 32,002/96,036). The proportion of males was 50.5% (793,382/1,572,152) overall, but that among patients with chronic ACSCs was 59.9% (7,338/12,250). The proportion of patients with acute ACSCs was higher in July (9,672, 13.9%) and August (10,291, 14.8%), while the proportions of patients with chronic ACSCs (1,986, 16.2%) and preventable ACSCs (10,021, 10.4%) were highest in January. As for the time of day when patients were transported by ambulance, the proportion of patients transported in daytime was small (30.3%, 475,802/1,572,152) overall, but was larger among patients with chronic ACSCs (53.6%, 6,561/12,250). Regarding the outcome after treatment at the emergency department, 42.6% (670,392/1,572.152) of patients were hospitalized, while 82.3% (10,079/12,250) of patients with chronic ACSCs were hospitalized. The most common disease in patients with acute ACSCs was volume depletion [E86, 63.9% (*n* = 48,416)], followed by acute tubule-interstitial nephritis [N10, 11.6% (*n* = 8,788)] (Supplementary Table 1). The most common disease in patients with chronic ACSCs was heart failure [I50, 23.1% (*n* = 24,507)], followed by essential hypertension [I10, 17.2% (*n* = 18,309)] (Supplementary Table 2). The most common disease in patients with preventable ACSCs was bacterial pneumonia [J15.9, 56.6% (*n* = 8,217)], followed by pneumoniae due to *Streptococcus pneumoniae* [J13, 11.3% (*n* = 1,647)] (Supplementary Table 3).

**Table 1:** Patient characteristics among all cohorts and the propensity score-matched cohort.

	**Total**		**Not ACSC**		**Acute ACSC**		**Chronic ACSC**		**Preventable ACSC**	
	**(*n =* 1,572,152)**		**(*n =* 1,394,245)**		**(*n =* 69,621)**		**(*n =* 12,250)**		**(*n =* 96,036)**	
Age, year, median (IQR)	71	(46–82)	70	(46–81)	76	(60–84)	80	(72–87)	66	(3–81)
**Age group, *n* (%)**										
Child (0–19 years)	1,30,886	(8.3)	96,408	(6.9)	2,099	(3.0)	377	(3.1)	32,002	(33.3)
Adult (20–64 years)	5,06,300	(32.2)	4,72,086	(33.9)	17,969	(25.8)	1,403	(11.5)	14,842	(15.5)
Elderly (≥65 years)	9,34,966	(59.5)	8,25,751	(59.2)	49,553	(71.2)	10,470	(85.5)	49,192	(59.5)
Male, *n* (%)	7,93,382	(50.5)	7,01,103	(50.3)	35,244	(50.6)	7,338	(59.9)	49,697	(51.7)
**Seasonality**										
January	1,49,757	(9.5)	1,32,703	(9.5)	5,047	(7.2)	1,986	(16.2)	10,021	(10.4)
February	1,25,758	(8.0)	1,11,591	(8.0)	4,348	(6.2)	1,362	(11.1)	8,457	(8.8)
March	1,23,678	(7.9)	1,10,134	(7.9)	4,505	(6.5)	1,055	(8.6)	7,984	(8.3)
April	1,18,228	(7.5)	1,05,285	(7.6)	4,590	(6.6)	900	(7.3)	7,453	(7.8)
May	1,22,705	(7.8)	1,09,232	(7.8)	5,109	(7.3)	844	(6.9)	7,520	(7.8)
June	1,22,922	(7.8)	1,08,447	(7.8)	5,887	(8.5)	719	(5.9)	7,869	(8.2)
July	1,47,165	(9.4)	1,28,461	(9.2)	9,672	(13.9)	853	(7.0)	8,179	(8.5)
August	1,49,983	(9.5)	1,31,546	(9.4)	10,291	(14.8)	889	(7.3)	7,257	(7.6)
September	1,25,687	(8.0)	1,11,764	(8.0)	6,117	(8.8)	822	(6.7)	6,984	(7.3)
October	1,26,163	(8.0)	1,12,525	(8.1)	5,011	(7.2)	842	(6.9)	7,785	(8.1)
November	1,23,697	(7.9)	1,10,735	(7.9)	4,321	(6.2)	842	(6.9)	7,799	(9.1)
December	1,36,409	(8.7)	1,21,822	(8.7)	4,723	(6.8)	1,136	(9.3)	8,728	(9.1)
**Time zone**										
Daytime (9:00–17:59)	4,75,802	(30.3)	4,15,797	(29.8)	27,253	(39.1)	6,561	(53.6)	26,191	(27.3)
Night time (0:00–8:59, 18:00–23:59)	10,96,350	(69.7)	9,78,448	(70.2)	42,368	(60.9)	5,689	(46.4)	69,845	(72.7)
**Outcome at emergency department**										
Hospitalization	6,70,392	(42.6)	5,82,641	(41.8)	33,508	(48.1)	10,079	(82.3)	44,164	(46.0)
Discharge home	8,59,540	(54.7)	7,71,145	(55.3)	35,301	(50.7)	1,959	(16.0)	51,135	(53.2)
Inter-hospital transfer	21,788	(1.4)	20,198	(1.4)	786	(1.1)	200	(1.6)	604	(2.8)
Dead	20,279	(1.3)	20,114	(1.4)	24	(0.0)	12	(1.2)	129	(0.1)
No visiting	153	(0.0)	147	(0.0)	2	(0.0)	0	(0)	4	(0.0)

*IQR, interquartile; ACSS*.

**Figure 1:**
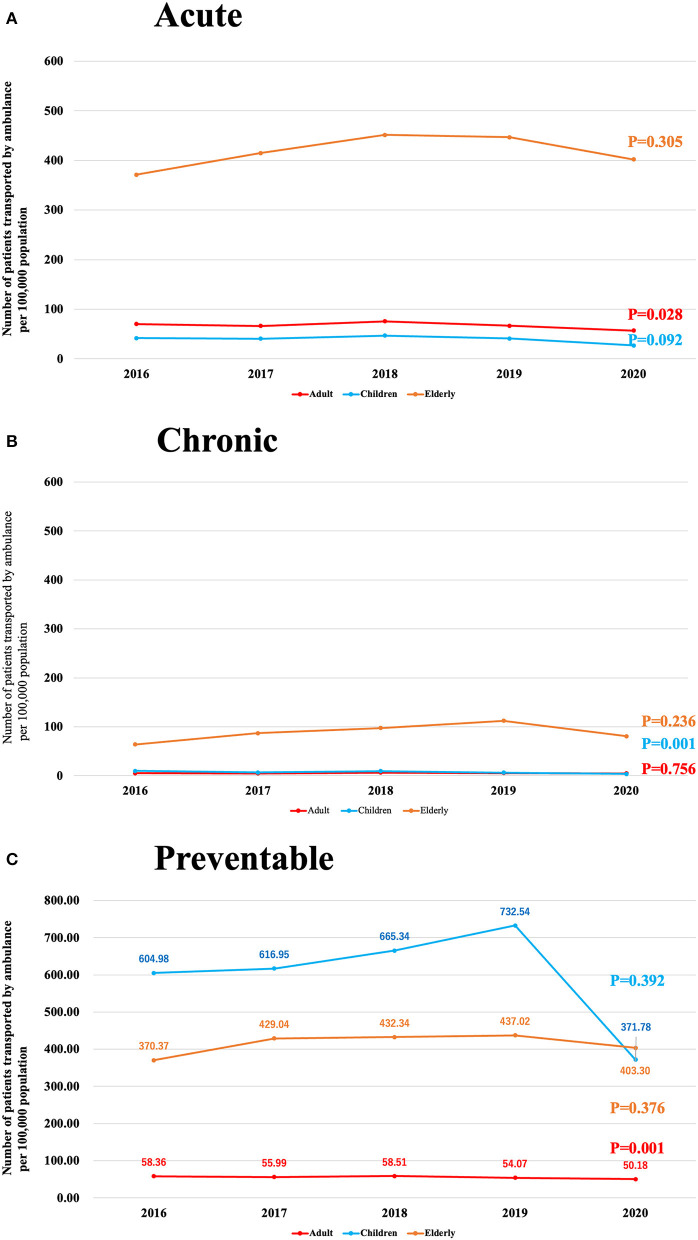
**(A)**, Trends over time in the number of acute ACSCs patients transported by ambulance per 100,000 population. **(B)**, Trends over time in the number of chronic ACSCs patients transported by ambulance per 100,000 population. **(C)**, Trends over time in the number of preventable ACSCs patients transported by ambulance per 100,000 population.

Figure 1A shows the trend in the number of patients with acute ACSCs per 100,000 population by age group. No change was observed in children (*P* = 0.092) or elderly individuals (*P* = 0.305), while the number of these patients decreased among adults (*P* = 0.028).

Figure 1B shows the trend in the number of patients with chronic ACSCs per 100,000 population by age group. No change was observed in adults (*P* = 0.756) or elderly individuals (*P* = 0.236), but the number of these patients decreased among children (*P* = 0.001).

Figure 1C shows the trend in the number of patients with preventable ACSCs per 100,000 population by age group. No change was observed in adults (*P* = 0.001) or elderly individuals (*P* = 0.376), but among children, the number increased until 2019 (732.5) and then decreased to 371.8 in 2020 (*P* = 0.392).

Table 2 shows the characteristics and outcomes of 670,392 hospitalized patients who were transported by ambulance. The proportion of males was 53.7% (359,718/670,392) overall, but was 60.5% (6,102/10,079) in patients with chronic ACSCs. The proportion of patients with acute ACSCs was higher in July (3,928, 11.7%) and August (4,150, 12.4%), while the proportions of patients with chronic ACSCs (1,367, 13.6%) and preventable ACSCs (4,894, 11.1%) were highest in January. Overall, fewer patients were transported during the daytime (278,912, 41.6%), but more were transported during the daytime among patients with chronic ACSCs (5,856, 58.1%). In terms of the outcome at 21 days after hospital admission, 26.5% (176,577/670,392) of all hospitalized patients remained hospitalized and 7.0% (46,504/670,392) had died, while 29.9% (3,009/10,079) of patients with chronic ACSCs remained hospitalized and 8.7% (871/10,079) had died.

**Table 2:** Unnecessary ambulance use with or without telephone triage service.

	**Total**		**Not ACSS**		**Acute ACSS**		**Chronic ACSS**		**Preventable ACSS**	
	**(*n =* 670,392)**		**(*n =* 582,641)**		**(*n =* 33,508)**		**(*n =* 10,079)**		**(*n =* 44,164)**	
Age, years, median (IQR)	77	(64–84)	77	(64–84)	79	(70–86)	81	(74–87)	76	(55–85)
**Age group, *n* (%)**										
Child (0–19 years)	19,306	(2.9)	12,065	(2.1)	163	(0.5)	118	(1.2)	6,960	(15.8)
Adult (20–64 years)	7,913	(1.2)	7,335	(1.3)	174	(0.5)	56	(0.6)	348	(0.8)
Elderly (≥65 years)	18,135	(2.7)	17,048	(2.9)	567	(1.7)	57	(0.6)	463	(1.0)
Male, *n* (%)	3,59,718	(53.7)	3,13,011	(53.7)	16,883	(50.4)	6,102	(60.5)	23,722	(53.7)
**Seasonality**										
January	62,731	(9.4)	53,947	(9.3)	2,523	(7.5)	1,367	(13.6)	4,894	(11.1)
February	52,878	(7.9)	45,777	(7.9)	2,191	(6.5)	943	(9.4)	3,967	(9.0)
March	53,475	(8.0)	46,577	(8.0)	2,323	(6.9)	871	(8.6)	3,704	(8.4)
April	51,485	(7.7)	44,831	(7.7)	2,408	(7.2)	782	(7.8)	3,464	(7.8)
May	52,782	(7.9)	46,036	(7.9)	2,536	(7.6)	745	(7.4)	3,465	(7.8)
June	52,398	(7.8)	45,518	(7.8)	2,871	(8.6)	645	(6.4)	3,364	(7.6)
July	59,946	(8.9)	51,850	(8.9)	3,928	(11.7)	766	(7.6)	3,402	(7.7)
August	60,569	(9.0)	52,380	(9.0)	4,150	(12.4)	798	(7.9)	3,241	(7.3)
September	53,715	(8.0)	46,781	(8.0)	3,077	(9.2)	726	(7.2)	3,131	(7.1)
October	55,704	(8.3)	48,712	(8.4)	2,672	(8.0)	750	(7.4)	3,570	(8.1)
November	55,154	(8.2)	48,274	(8.3)	2,395	(7.1)	752	(7.5)	3,733	(8.5)
December	59,555	(8.9)	51,958	(8.9)	2,434	(7.3)	934	(9.3)	4,229	(9.6)
**Time zone**										
Daytime (9:00–17:59)	2,78,912	(41.6)	2,38,766	(41.0)	16,462	(49.1)	5,856	(58.1)	17,828	(40.4)
Night time (0:00–8:59, 18:00–23:59)	3,91,480	(58.4)	3,43,875	(59.0)	17,046	(50.9)	4,223	(41.9)	26,336	(59.6)
**Outcome at emergency department**										
Hospitalization	1,76,577	(26.5)	1,54,290	(26.6)	9,152	(27.4)	3,009	(29.9)	10,126	(23.0)
Discharge home	4,12,509	(61.8)	3,54,246	(61.1)	21,579	(64.7)	5,753	(57.2)	30,931	(70.2)
Inter-hospital transfer	31,598	(4.7)	28,275	(4.9)	1,573	(4.7)	425	(4.2)	1,325	(3.0)
Dead	46,504	(7.0)	42,927	(7.4)	1,052	(3.2)	871	(8.7)	1,654	(3.8)
Unknown	3,204	(0.0)	147	(0.0)	2	(0.0)	0	(0)	4	(0.0)

*IQR, interquartile; ACSS*.

## Discussion

In this study, we revealed the characteristics and outcomes of patients with ACSCs using a population-based registry for emergency patients transported by ambulance in Osaka, Japan. Patients with chronic ACSCs were predominantly elderly, while patients with preventable ACSCs were polarized between children and the elderly. Patients with acute ACSCs were more frequently transported in the summer, while those with chronic ACSCs and preventable ACSCs were more frequently transported in the winter. Although there was no change over time in adults and children, there was a marked decrease among the elderly with preventable ACSCs after 2020. An increase in the number of patients transported by ambulance due to ACSCs, which do not cause serious illness if the patients properly manage their daily medications and physical condition, would have a serious impact on the emergency medical system. Therefore, it is necessary to reveal the actual status of patients with ACSCs among those transported by ambulance. Japan is one of the countries with high proportion of elderly people in the world, and there has been no studies using population-based data in Japan, which makes this study highly novel. This study, which revealed the characteristics and outcomes of patients with ACSCs using population-based data, is useful not only for further understanding the reality of patients with ACSCs, but also for considering health policies in relation to ACSCs.

In this study, volume depletion and urinary tract infection (e.g., acute pyelonephritis) were common among patients with acute ACSCs. Regarding age group, the proportion of elderly patients in this population was higher than that of emergency patients due to sudden illness other than ACSCs. ACSCs have been reported in many countries, mostly in European countries and Brazil (10–20). However, the seasonality of acute ACSCs has not been revealed thus far. In Japan, temperatures are rising with global warming, especially in summer. Thus, deaths due to heat stroke have been reported (21). Elderly individuals are not only unable to adequately regulate their body temperature due to aging, but also require strict fluid control due to existing diseases, such as heart failure and renal failure. These factors may make the elderly more susceptible to dehydration in summer. Therefore, fluid control in the elderly is important in daily practice and daily care, and appropriate fluid control is required for the prevention of dehydration and urinary tract infection.

Second, patients with chronic ACSCs included a higher proportion of patients with heart failure and essential hypertension, a higher proportion of elderly individuals, and a higher proportion of males. An observational study in Switzerland reported that heart failure and chronic obstructive pulmonary disease (COPD) were the main causes of hospitalization for elderly patients with ACSCs (22). In an observational study in Finland, older age and living alone were associated with hospitalization for patients with ACSCs (23). In addition, an observational study in Canada reported that COPD, diabetes mellitus (DM), and higher comorbidity index were associated with frequent ER visits in elderly patients with ACSCs (16). In chronic diseases such as heart failure and COPD, daily management of the disease is important to prevent the exacerbation of symptoms. Some previous studies have shown that men have higher medication adherence (24, 25). If men have better medication compliance, other factors may be associated with the finding that more male patients with chronic ACSCs were transported to emergency departments in this study. This may be related to socioeconomic factors, such as living alone or poverty. Patients with chronic ACSCs were more likely to be transported by ambulance during the daytime. Elderly individuals tend to use ambulances as a means of visiting emergency departments (26), and patients with chronic ACSCs may be dependent on ambulance transportation as a means of visiting hospitals. Ambulances should be used for emergency situations (e.g., traffic accidents and cardiopulmonary arrest) and it may be necessary to establish transportation service with an inexpensive subsidized price as an alternative to ambulances for elderly individuals.

In this study, the number of patients with preventable ACSCs was high in both children and elderly individuals, and the most common diseases were bacterial pneumonia, pneumococcal pneumonia, and influenza. In Japan, the elderly individuals can be vaccinated against these diseases free of charge under the public health system; however, children are not covered by the health system and their parents must pay the full cost of vaccination (27). It is very important to prevent influenza infection in children because complications of influenza-induced encephalopathy have a significant neurological impact. Therefore, if vaccination of children can be encouraged by making influenza vaccination a part of the public health system, as is the case with the elderly population, the number of pediatric patients with influenza may decrease and the number of emergency pediatric patients with influenza may also decrease. In addition, no change was observed in adults or elderly individuals over time, but there was a marked decrease among children in 2020. The novel corona virus confirmed in Wuhan, China, in 2019 had a profound impact around the world (28), and people embraced new lifestyles, including wearing masks and using alcohol to disinfect their hands as measures to prevent infection. The acceptance of these measures by many parents and children may have prevented the spread of infectious diseases, including influenza, and reduced the number of pediatric patients who were transported by ambulance.

### Limitations

The present study was associated with some limitations. First, it was no possible to assess the patients with ACSCs who visited hospitals and clinics on their own, because this study included patients transported by ambulance. Second, because the patient transport by ambulance due to ACSCs is a rare occurrence, a Poisson distribution was assumed and Poisson regression analysis was performed in this study. On the other hand, the Poisson assumption may cause overdispersion problem because the variance is not able to determine in the Poisson distribution. Third, the generalizability of this study is high because we analyzed a dataset collected over several years in metropolitan area of Japan including urban and rural areas. However, this study was an analysis of data from Japan and may lack validity outside of Japan. Next, because this was a retrospective observational study, we were not able to reveal other variables that were not collected in the ORION system.

## Conclusion

In this study, patients with chronic ACSCs were predominantly elderly, while patients with preventable ACSCs were polarized between children and the elderly. Among patients with preventable ACSCs, there was no change over time in adults and children, but there was a marked decrease among the elderly after 2020. We will continue to evaluate the impact of factors such as the increase of the elderly and the COVID-19 pandemic on patient transport by ambulance due to ACSCs.

## Data Availability Statement

The data analyzed in this study is subject to the following licenses/restrictions: The data that support the findings of this study are available from the Osaka Prefectural Government, but the availability of these data are restricted. Data cannot be shared publicly because of the Protection Ordinance for Personal Information in Osaka Prefecture. Requests to access these datasets should be directed to Osaka Prefectural Government, iryotaisaku-g02@sbox.pref.osaka.lg.jp.

## Author Contributions

YK, AK, and YY carried out the study protocol. YK, AK, and TK undertook the statistical analysis and graphical representation of the data. YK, TK, and JO revised the draft. All authors participated in the completion of the study and contributed to and approved the final manuscript.

## Funding

This study was supported by the Japan Society for the Promotion of Science KAKENHI (Grant No. JP21K09071).

## Conflict of Interest

The authors declare that the research was conducted in the absence of any commercial or financial relationships that could be construed as a potential conflict of interest.

## Publisher's Note

All claims expressed in this article are solely those of the authors and do not necessarily represent those of their affiliated organizations, or those of the publisher, the editors and the reviewers. Any product that may be evaluated in this article, or claim that may be made by its manufacturer, is not guaranteed or endorsed by the publisher.

## References

[1] World Health Organization. Integrated Care for Older People. Available online at: https://apps.who.int/iris/bitstream/handle/10665/326295/WHO-HIS-SDS-2018.44-eng.pdf (accessed March 1, 2022).

[2] Ambulance Service Planning Office the Fire Disaster Management Agency. The effect of first aid for emergency patients in 2021. Available online at: https://www.fdma.go.jp/publication/rescue/items/kkkg_r03_01_kyukyu.pdf (accessed March 1, 2022).

[3] Ministry Ministry of Health Labour and Welfare of Japan. Medical Facility Survey in 2021. Available online at: https://www.mhlw.go.jp/toukei/saikin/hw/iryosd/m21/dl/is2103_01.pdf (accessed March 1, 2022).

[4] Katayama Y Kitamura T Kiyohara K Iwami T Kawamura T Hayashida S. Factors associated with the difficulty in hospital acceptance at the scene by emergency medical service personnel: a population-based study in Osaka City, Japan. BMJ Open. (2016) 6:e013849. 10.1136/bmjopen-2016-013849 27798040 PMC5093624

[5] Bardsley M Blunt I Davies S Dixon J. Is secondary preventive care improving? Observational study of 10-year trends in emergency admissions for conditions amenable to ambulatory care. BMJ Open. (2013) 3:e002007. 10.1136/bmjopen-2012-002007 23288268 PMC3549201

[6] Okamoto J Katayama Y Kitamura T Sado J Nakamura R Kimura N. Profile of the ORION (Osaka emergency information Research Intelligent Operation Network system) between 2015 and 2016 in Osaka, Japan: a population-based registry of emergency patients with both ambulance and in-hospital records. Acute Med Surg. (2019) 6:12–24. 10.1002/ams2.371 30651993 PMC6328924

[7] Katayama Y Kitamura T Kiyohara K Iwami T Kawamura T Izawa J. Improvements in patient acceptance by hospitals following the introduction of a smartphone app for the emergency medical service system: a population-based before-and-after observational study in Osaka City, Japan. JMIR Mhealth Uhealth. (2017) 5:e134. 10.2196/mhealth.8296 28893725 PMC5616023

[8] Osaka, Prefectural Government,. The National Census in Osaka, 2020. Available online at: https://www.pref.osaka.lg.jp/attach/1891/00201713/R2kokutyo_osakahu_kakuhou_toukeihyo.pdf (accessed March 1, 2022).

[9] von Elm E Altman DG Egger M Pocock SJ Gøtzsche PC Vandenbroucke JP. The Strengthening the Reporting of Observational Studies in Epidemiology (STROBE) statement: guidelines for reporting observational studies. Lancet. (2007) 370:1453–7. 10.1016/S0140-6736(07)61602-X 18064739

[10] Silva SS Pinheiro LC Loyola Filho AI. Spatial analysis of factors associated with hospitalizations for ambulatory care sensitive conditions among old adults in Minas Gerais State. Rev Bras Epidemiol. (2021) 24:e210037. 34133703 10.1590/1980-549720210037

[11] Phillips KG Wishengrad JS Houtenville AJ. Ambulatory care sensitive conditions among all-payer claimants with intellectual and developmental disabilities. Am J Intellect Dev Disabil. (2021) 126:203–15. 10.1352/1944-7558-126.3.203 33910241

[12] Goldfeld S Paton K Lei S Perera P Hiscock H. Trends in rates and inequalities in paediatric admissions for ambulatory care sensitive conditions in Victoria, Australia (2003 to 2013). J Paediatr Child Health. (2021) 57:860–6. 10.1111/jpc.15338 33432713

[13] Godard-Sebillotte C Strumpf E Sourial N Rochette L Pelletier E Vedel I. Avoidable hospitalizations in persons with dementia: a population-wide descriptive study (2000-2015). Can Geriatr J. (2021) 24:209–21. 10.5770/cgj.24.486 34484504 PMC8390329

[14] Carneiro VSM Vila V Vieira M. Trends in pediatric hospitalizations for ambulatory care sensitive respiratory diseases in Brazil. Public Health Nurs. (2021) 38:106–14. 10.1111/phn.12818 33043515

[15] Lumme S Manderbacka K Arffman M Karvonen S Keskimaki I. Cumulative social disadvantage and hospitalisations due to ambulatory care-sensitive conditions in Finland in 2011–2013: a register study. BMJ Open. (2020) 10:e038338. 10.1136/bmjopen-2020-038338 32847920 PMC7451287

[16] Dufour I Chiu Y Courteau J Chouinard MC Dubuc N Hudon C. Frequent emergency department use by older adults with ambulatory care sensitive conditions: a population-based cohort study. Geriatr Gerontol Int. (2020) 20:317–23. 10.1111/ggi.13875 32017348 PMC7187263

[17] Rodrigues MM Alvarez AM Rauch KC. Trends in hospitalization and mortality for ambulatory care sensitive conditions among older adults. Rev Bras Epidemiol. (2019) 22:e190010. 10.1590/1980-549720190010 30892473

[18] Silva M Oliveira VDS Pinto PMA Razia PFS Caixeta ACL Aquino É C. Trends of hospitalizations for ambulatory care-sensitive cardiovascular conditions in the municipality of Senador Canedo, Goiás, Brazil, 2001-2016. Epidemiol Serv Saude. (2019) 28:e2018110. 10.5123/S1679-49742019000100018 30970072

[19] Paul MC Dik JH Hoekstra T van Dijk CE. Admissions for ambulatory care sensitive conditions: a national observational study in the general and COPD population. Eur J Public Health. (2019) 29:213–9. 10.1093/eurpub/cky182 30212895 PMC6426039

[20] Mariano T Nedel FB. Hospitalization for Ambulatory Care Sensitive Conditions in children under five years old in Santa Catarina State, Brazil, 2012: a descriptive study. Epidemiol Serv Saude. (2018) 27:e2017322. 30281714 10.5123/S1679-49742018000300006

[21] Ministry Ministry of Health Labour and Welfare of Japan. Trends Over Time in the Number of Deaths Due to Heatstroke in Japan. Available online at: https://www.mhlw.go.jp/toukei/saikin/hw/jinkou/tokusyu/necchusho19/dl/nenrei.pdf (accessed March 1, 2022).

[22] Gygli N Zúñiga F Simon M. Regional variation of potentially avoidable hospitalisations in Switzerland: an observational study. BMC Health Serv Res. (2021) 21:849. 10.1186/s12913-021-06876-5 34419031 PMC8380390

[23] Partanen VM Arffman M Manderbacka K Keskimäki I. Mortality related to ambulatory care sensitive hospitalisations in Finland. Scand J Public Health. (2020) 48:839–46. 10.1177/1403494820944722 32755271

[24] Hussain S Jamal SZ Qadir F. Medication adherence in post myocardial infarction patients. J Ayub Med Coll Abbottabad. (2018) 30:552–7. 30632336

[25] Rolnick SJ Pawloski PA Hedblom BD Asche SE Bruzek RJ. Patient characteristics associated with medication adherence. Clin Med Res. (2013) 11:54–65. 10.3121/cmr.2013.1113 23580788 PMC3692389

[26] Durant E Fahimi J. Factors associated with ambulance use among patients with low-acuity conditions. Prehosp Emerg Care. (2012) 16:329–37. 10.3109/10903127.2012.670688 22494108

[27] National institute of infectious disease in Japan. The List of Vaccines Available in Japan. Available online at: https://www.niid.go.jp/niid/ja/vaccine-j/249-vaccine/589-atpcs003.html (accessed March 1, 2022).

[28] Wang C Horby PW Hayden FG Gao GF. A novel coronavirus outbreak of global health concern. Lancet. (2020) 395:470–3. 10.1016/S0140-6736(20)30185-9 31986257 PMC7135038

